# Two Cases of Intentional Sodium Nitrite Poisoning With Contrasting Outcomes: The Critical Role of Early Recognition and Treatment

**DOI:** 10.7759/cureus.113663

**Published:** 2026-07-30

**Authors:** Kinga Lichtarska, Sviatlana Filitaryna, Monika Wiatr, Magda Marciniak, Michał Gutowski, Monika Kałwak, Aleksandra Foryś, Wojciech Wojas, Cezary Orzechowski, Justyna Żabicka, Patryk Dłubis

**Affiliations:** 1 Department of Internal Medicine, St. Elizabeth’s Hospital, LUX MED Group, Warsaw, POL; 2 Department of Internal Medicine, Dr. Tytus Chałubiński Specialist Hospital, Radom, POL; 3 Department of Medicine, Medical University of Warsaw, Warsaw, POL; 4 Department of Internal Medicine, Independent Public Health Care Facility of the Ministry of the Interior and Administration named after Sergeant Grzegorz Załoga, Katowice, POL; 5 Department of Internal Medicine, St. Barbara Provincial Specialist Hospital No. 5, Sosnowiec, POL; 6 Department of Medicine, Maria Skłodowska-Curie Medical Academy, Warsaw, POL; 7 Department of Internal Medicine, Municipal Hospital in Gliwice, Gliwice, POL

**Keywords:** antidote, case report, emergency medicine, intensive care, methemoglobinemia, methylene blue, poisoning, sodium nitrite, suicide attempt, toxicology

## Abstract

Intentional sodium nitrite poisoning may result in severe methemoglobinemia and life-threatening tissue hypoxia. We present two cases of intentional sodium nitrite ingestion with markedly different clinical outcomes, both managed at the same center and treated with methylene blue at different stages of clinical deterioration, thereby illustrating the potential importance of early recognition and treatment timing.

The first patient was a 31-year-old man without a significant medical history who was found unconscious in his apartment after an unknown interval following ingestion; therefore, the time to emergency medical intervention could not be determined. He remained in cardiac arrest upon admission, with ongoing advanced life support, mechanical ventilation, and mechanical chest compressions. Methylene blue was administered intravenously immediately upon admission to the Toxicology Unit in two doses of 2 mg/kg (170 mg each) given 15 minutes apart, corresponding to a total cumulative dose of 4 mg/kg (340 mg). Initial laboratory investigations revealed an extremely high methemoglobin level of 97% (typical reference value: <2%), profound acidosis (pH 6.684), severe hyperlactatemia (29 mmol/L), and critically impaired oxygenation. Return of spontaneous circulation was not achieved, and the patient was pronounced dead 40 minutes after admission to the Toxicology Unit.

The second patient was a 22-year-old woman with a history of personality disorder who ingested 7.5 g of sodium nitrite and promptly informed her family, enabling rapid activation of Emergency Medical Services (EMS). Methylene blue was administered intravenously approximately 50 minutes after ingestion in two doses of 2 mg/kg (160 mg each) given 15 minutes apart, corresponding to a total cumulative dose of 4 mg/kg (320 mg). Her initial methemoglobin concentration was 66.1%, and antidotal treatment resulted in rapid clinical and laboratory improvement. Despite requiring prolonged intensive care and treatment for pneumonia, she was discharged after 22 days without neurological sequelae.

These cases highlight the critical importance of early recognition and prompt initiation of antidotal therapy in sodium nitrite poisoning. The markedly different outcomes observed in two patients exposed to the same toxic agent suggest that prolonged untreated tissue hypoxia may have a greater influence on prognosis than the absolute methemoglobin concentration alone. Although no firm conclusions can be drawn from two cases, the findings suggest that early methylene blue administration combined with intensive supportive care may be associated with favorable outcomes even in severe poisoning.

## Introduction

Sodium nitrite is an inorganic compound widely used in the food industry as a preservative, as well as in chemical manufacturing, laboratory analysis, and the production of azo dyes [1,2]. Although accidental exposure is uncommon, intentional sodium nitrite poisoning has become an emerging toxicological concern in recent years, largely due to the widespread online availability of highly concentrated preparations and information regarding their lethal potential. This trend has led to a noticeable rise in deliberate self-poisonings involving sodium nitrite, posing significant challenges for timely recognition and management in emergency settings [1,3-5].

The toxic effects of sodium nitrite are primarily mediated through oxidation of hemoglobin iron from the ferrous (Fe²⁺) to the ferric (Fe³⁺) state, resulting in the formation of methemoglobin. Unlike normal hemoglobin, methemoglobin is incapable of effectively binding and transporting oxygen [6-9]. Furthermore, its presence increases the oxygen affinity of the remaining functional hemoglobin molecules, shifting the oxyhemoglobin dissociation curve to the left and thereby impairing oxygen release to peripheral tissues. Consequently, tissue hypoxia may develop despite adequate oxygen delivery to the lungs. Furthermore, evaluating the extent of this hypoxia can be clinically challenging, as oxygen saturation measured by pulse oximetry may be unreliable in severe methemoglobinemia [6,7,10].

Under physiological conditions, small amounts of methemoglobin are continuously formed and reduced by enzymatic pathways within erythrocytes, maintaining methemoglobin concentrations below 1-2% of total hemoglobin. Following exposure to potent oxidizing agents such as sodium nitrite, however, these compensatory mechanisms may become overwhelmed, leading to rapid accumulation of methemoglobin and impaired oxygen transport [8,10]. Additionally, clinical management may be influenced by underlying enzymatic defects such as cytochrome b5 reductase or G6PD deficiency, although G6PD deficiency is a relative contraindication for methylene blue due to the potential risk of hemolysis, a single dose is generally acceptable in severe cases [7].

The clinical manifestations of methemoglobinemia are closely related to methemoglobin concentration. Cyanosis typically develops at levels around 10-15%, while concentrations exceeding 20-50% may result in dizziness, syncope, dyspnea, exercise intolerance, fatigue, headache, and weakness. Severe poisoning, usually associated with methemoglobin levels above 50%, may lead to dysrhythmia, seizure, coma, and metabolic acidosis [7,9]. Methemoglobin concentrations exceeding 70% are generally considered potentially fatal [7,9,10].

Methylene blue remains the antidotal treatment of choice for clinically significant methemoglobinemia [8,11-13]. Acting as an artificial electron carrier, it accelerates the reduction of methemoglobin back to functional hemoglobin through the NADPH-dependent pathway [8,11]. However, the effectiveness of treatment depends largely on prompt recognition of the condition and rapid initiation of therapy before irreversible hypoxic organ injury develops [12,14,15].

We present two cases of intentional sodium nitrite poisoning with markedly different clinical outcomes despite exposure to the same toxic agent. These cases highlight the critical importance of early diagnosis and timely administration of antidotal therapy in determining patient prognosis.

## Case presentation

Case 1

A 31-year-old man with no history of chronic medical or psychiatric illness was brought to the hospital by Emergency Medical Services (EMS) after being found unconscious and cyanotic on the floor of his apartment. An empty package of sodium nitrite and a suicide note were found next to the patient. The exact interval between ingestion and discovery remained unknown. Consequently, the time from ingestion to emergency medical intervention could not be determined.

Due to respiratory insufficiency, the patient was intubated in the prehospital setting and placed on mechanical ventilation with 100% oxygen. Despite maximal oxygen supplementation, profound cyanosis persisted, suggesting severe impairment of oxygen transport and utilization. Within minutes, the patient developed bradycardia followed by cardiac arrest with asystole. Advanced cardiopulmonary resuscitation was initiated, and a mechanical chest compression device was employed during transport to the Toxicology Unit.

Upon admission, the patient remained in an agonal state during ongoing resuscitation efforts. Central and peripheral cyanosis persisted, arterial blood pressure was unmeasurable, and the pupils were dilated and nonreactive. Mechanical chest compressions had already been ongoing for approximately 75 minutes.

Given the suspicion of severe methemoglobinemia, methylene blue was administered intravenously immediately upon admission to the Toxicology Unit, although the time elapsed since ingestion remained unknown. The patient received two doses of 2 mg/kg (170 mg each), administered 15 minutes apart, corresponding to a total cumulative dose of 4 mg/kg (340 mg). No clinical improvement was observed. Asystole, cyanosis, and the absence of effective circulation persisted. Mechanical ventilation, cardiopulmonary resuscitation, intravenous fluid therapy, and repeated doses of epinephrine were continued.

Initial laboratory findings on admission are summarized in Table 1. Arterial blood gas analysis revealed extremely severe methemoglobinemia (Met-Hb 97%), near-complete absence of arterial oxygenation (pO₂ 0.2 mmHg), profound mixed acidosis (pH 6.684; HCO₃⁻ 8.9 mmol/L), markedly elevated lactate concentration (29 mmol/L), and a substantial base deficit (-27.5 mmol/L). Additional findings included hypercapnia (pCO₂ 76.6 mmHg), hyperkalemia (7.6 mmol/L), mildly elevated serum creatinine (1.58 mg/dL) with normal blood urea levels, and normal liver transaminase activity. Toxicological screening was negative for ethanol, benzodiazepines, phenobarbital, valproic acid, carbamazepine, and acetaminophen.

**Table 1:** Laboratory findings on admission. * The performing laboratory did not state a reference range; the typical reference value is <2% [6]. **The toxicology screening included benzodiazepines, phenobarbital, valproic acid, carbamazepine, and acetaminophen in Case 1, and cocaine, amphetamine, methamphetamine, MDMA, opiates, cannabinoids, and mephedrone in Case 2. ALT: alanine aminotransferase; AST: aspartate aminotransferase; HCO₃⁻: bicarbonate; MCH: mean corpuscular hemoglobin; MCHC: mean corpuscular hemoglobin concentration; MCV: mean corpuscular volume; pCO₂: partial pressure of carbon dioxide; pH: potential of hydrogen; pO₂: partial pressure of oxygen; RBC: red blood cell count; WBC: white blood cell count

Parameter	Case 1	Case 2	Reference range	Unit
Arterial blood gas analysis				
Methemoglobin	97.0	66.1	<2*	%
pH	6.684	7.544	7.350 – 7.450	
pO₂	0.2	62.8	75.0 – 100.0	mmHg
pCO₂	76.6	22.3	32.0 – 45.0	mmHg
HCO₃⁻	8.9	19.2	22 - 28	mmol/L
Lactate	29.0	4.2	0.5 – 1.6	mmol/L
Base excess	-27.5	-1.3	-2.0 – 2.0	mmol/L
Complete blood count				
WBC	8.27	12.75	4.23 – 10.00	10³/µL
RBC	4.24	4.79	4.63 – 6.08	10^6^/uL
MCV	105.7	89.6	79.0 – 92.2	fL
MCH	31.1	29.6	25.7 – 32.2	pg
MCHC	29.5	33.1	32.3 – 36.5	g/dL
Hemoglobin	13.2	14.2	13.7 – 17.5	g/dL
Hematocrit	44.8	42.9	40.1 – 51.0	%
Platelet count	218	266	140 - 400	10³/µL
Venous blood biochemistry				
Potassium	7.60	4.00	3.50 – 5.10	mmol/L
Creatinine	1.58	0.82	0.80 – 1.20	mg/dL
AST	28	30	10 - 37	U/L
ALT	28	40	10 - 37	U/L
Toxicology				
Blood ethanol	Negative	Negative		
Toxicology screening**	Negative	Negative		

Despite aggressive resuscitative measures and antidotal therapy, return of spontaneous circulation was not achieved. The patient was pronounced dead 40 minutes after admission to the Toxicology Unit, the immediate cause of death being refractory cardiac arrest with persistent asystole secondary to profound methemoglobinemia and severe tissue hypoxia.

Case 2

A 22-year-old woman with a history of personality disorder was admitted to the Toxicology Unit following the intentional ingestion of 7.5 g of sodium nitrite purchased online. After ingesting the substance, she informed her mother, who subsequently contacted EMS.

Upon EMS arrival, the patient was conscious, oriented, and able to answer questions appropriately. She was hemodynamically and respiratory stable at that time. However, progressive clinical deterioration was observed during transport to the hospital.

On admission, the patient was in critical condition. She exhibited worsening respiratory failure with bradypnea (five breaths/min), intermittent apnea, and cyanosis. Oxygen saturation was 65% despite supplemental oxygen therapy. Blood pressure remained within the normal range (116/80 mmHg), while sinus tachycardia of 120 beats/min was present. Non-contrast computed tomography of the head revealed no acute abnormalities.

Toxicological testing demonstrated severe methemoglobinemia, with a methemoglobin concentration of 66.1%. Arterial blood gas analysis showed hypoxemia, hypocapnia, and compensated metabolic acidosis. Toxicological screening was negative for ethanol, and urine testing did not detect common drugs of abuse, including cocaine, amphetamine, methamphetamine, MDMA, opiates, cannabinoids, or mephedrone.

Methylene blue was administered intravenously immediately upon admission, approximately 50 minutes after sodium nitrite ingestion. The patient received two doses of 2 mg/kg (160 mg each), administered 15 minutes apart, corresponding to a total cumulative dose of 4 mg/kg (320 mg). Simultaneously, the patient underwent endotracheal intubation and was placed on mechanical ventilation. Sedation was maintained with midazolam and propofol. Due to the development of hypotension (60/40 mmHg), intravenous norepinephrine infusion was started.

A rapid clinical response was observed after the second dose of methylene blue. Cyanosis resolved and oxygenation parameters progressively improved. Approximately 10 minutes after antidote administration, the methemoglobin concentration had decreased from 66.1% to 5.5%. Arterial blood gas parameters normalized within the following two hours. Vasopressor support was successfully discontinued after 12 hours.

The subsequent hospital course was complicated by the development of right-sided pneumonia requiring prolonged mechanical ventilation and antibiotic therapy. Empirical treatment was initiated with ceftriaxone and amikacin and later escalated to ceftazidime and vancomycin, resulting in a favorable clinical response. Chest imaging demonstrated reduced transparency of the right lung field, right pleural effusion, and atelectatic changes involving the middle lobe of the right lung.

The patient was successfully weaned from mechanical ventilation and extubated on hospital day 9. Because of persistent psychomotor agitation, sedation was continued for an additional four days. From day 13 onward, she remained fully conscious, with normal logical contact and no neurological abnormalities. During hospitalization, transient elevations of liver enzymes, international normalized ratio (INR), and creatine kinase levels, as well as mild anemia, were observed. All abnormalities improved before discharge. The patient was discharged home on hospital day 22 in good general condition, without respiratory or circulatory insufficiency and with no neurological sequelae. A detailed side-by-side comparison of the demographic, clinical, therapeutic, and outcome parameters for both cases is provided in Table 2.

**Table 2:** Clinical comparison of the presented sodium nitrite poisoning cases.

Parameter	Case 1	Case 2
Age and sex	31 years, Male	22 years, Female
Estimated ingested dose	Unknown	7.5 g
Time to presentation	Unknown	Approximately 50 minutes post-ingestion
Methemoglobin level	97.0%	66.1%
Treatment initiation	Immediately on admission (time post-ingestion unknown)	Approximately 50 minutes post-ingestion
Methylene blue dose	4 mg/kg (340 mg) total (two 2 mg/kg doses 15 min apart)	4 mg/kg (320 mg) total (two 2 mg/kg doses 15 min apart)
Complications	Cardiac arrest (asystole), severe mixed acidosis, refractory tissue hypoxia	Respiratory failure, hypotension, right-sided pneumonia, transient elevation of liver enzymes/INR
Outcome	Fatal	Full recovery (discharged home on day 22)

## Discussion

The present cases illustrate two markedly different clinical outcomes following intentional ingestion of the same toxic agent - sodium nitrite - and suggest that prognosis in severe methemoglobinemia may depend not only on the amount of toxin ingested but also on the time elapsed between exposure and initiation of specific treatment.

Clinical outcome in sodium nitrite poisoning is largely determined by the duration of tissue hypoxia [7,9]. Methemoglobin not only loses its ability to transport oxygen but also impairs oxygen release from the remaining functional hemoglobin molecules by shifting the oxyhemoglobin dissociation curve to the left [6,7,10]. Consequently, cellular metabolism rapidly shifts toward anaerobic pathways, resulting in progressive lactic acidosis and organ dysfunction [10].

The first patient presented with features of extremely advanced poisoning. Laboratory testing demonstrated near-complete loss of oxygen-carrying capacity (Met-Hb 97%), profound mixed acidosis, severe hyperlactatemia, and virtually absent arterial oxygenation. These findings strongly suggest that extensive tissue hypoxia and irreversible cellular injury had already occurred before treatment was initiated. In such circumstances, even prompt administration of methylene blue and aggressive resuscitative efforts may be ineffective, as restoration of hemoglobin function cannot reverse the consequences of prolonged hypoxic injury [14].

Conversely, despite severe clinical presentation and a methemoglobin concentration of 66.1%, the second patient experienced complete recovery following rapid initiation of treatment. Particularly noteworthy was the immediate response to methylene blue, with methemoglobin levels decreasing significantly within a short time of administration. Such a rapid response suggests that antidotal therapy was administered before the development of irreversible organ damage.

These cases also highlight the limitations of conventional methods used to assess oxygenation. In methemoglobinemia, pulse oximetry readings may not accurately reflect the degree of tissue hypoxia and may therefore underestimate the severity of impaired oxygen transport [11,13]. Consequently, clinical findings remain critically important. Persistent cyanosis despite supplemental oxygen therapy, the characteristic chocolate-brown appearance of blood, and rapidly worsening clinical status should prompt immediate consideration of methemoglobinemia [10,13,15,16].

It is also noteworthy that the second patient required prolonged intensive care despite successful reversal of methemoglobinemia. Her hospital course was complicated by pneumonia requiring extended mechanical ventilation and antibiotic therapy. Nevertheless, the final outcome was favorable, with complete neurological recovery and no persistent sequelae. This observation suggests that even severe sodium nitrite poisoning may have a good prognosis when tissue hypoxia is rapidly corrected and comprehensive supportive care is provided.

Several limitations should be considered when interpreting these observations. This report describes only two patients and therefore cannot establish causal relationships or identify independent prognostic factors. In Case 1, the amount ingested and the interval between ingestion, discovery, and treatment were unknown, limiting direct comparison with Case 2. Moreover, differences in the patients’ clinical condition at presentation, duration of hypoxia, ingested dose, and other individual factors may have contributed to the contrasting outcomes. Accordingly, the findings should be interpreted as illustrative clinical observations rather than evidence that treatment timing alone determines prognosis.

Beyond their clinical implications, these cases also highlight an emerging public health concern. The widespread online availability of sodium nitrite and increasing public awareness of its lethality have contributed to a growing number of intentional poisonings reported worldwide. Recent evidence indicates that discussions concerning sodium nitrite have increased substantially within online suicide-related communities, where users may exchange information regarding acquisition, preparation, and potentially lethal doses. This phenomenon has coincided with a marked rise in reported poisonings and fatalities in several countries. Given the high mortality associated with delayed diagnosis and treatment, increased awareness among healthcare professionals and appropriate regulatory measures aimed at restricting access to concentrated sodium nitrite preparations may help reduce the number of future fatalities [1-5].

## Conclusions

The present cases highlight the potential importance of the interval between sodium nitrite ingestion, diagnosis, and initiation of antidotal therapy in determining clinical outcome. Although both patients developed severe methemoglobinemia, their markedly different outcomes suggest that the duration of untreated tissue hypoxia may influence prognosis more strongly than the methemoglobin concentration measured at hospital admission. However, given the descriptive nature of this two-case report, no definitive conclusions regarding prognostic factors can be drawn. Early administration of methylene blue, together with intensive supportive care, may rapidly restore oxygen-carrying capacity and help prevent irreversible organ injury, whereas delayed treatment may be associated with profound tissue hypoxia, severe metabolic acidosis, cardiac arrest, and death.

These cases also underscore the importance of maintaining a high index of suspicion for methemoglobinemia in patients presenting with acute respiratory failure and cyanosis that does not improve despite oxygen supplementation. Persistent cyanosis, low oxygen saturation, and the lack of an expected response to oxygen therapy should prompt immediate evaluation for methemoglobinemia and urgent measurement of methemoglobin levels. Successful management of severe sodium nitrite poisoning requires not only prompt administration of methylene blue but also comprehensive supportive care, including airway protection, treatment of respiratory and circulatory failure, and management of potential complications. Given the increasing availability of sodium nitrite through online vendors and the growing number of reports describing its use in suicide attempts, sodium nitrite poisoning should be considered in the differential diagnosis of patients presenting with severe hypoxia of unclear etiology, particularly when accompanied by persistent cyanosis despite oxygen supplementation.
